# Transposon Reactivation in the Germline May Be Useful for Both Transposons and Their Host Genomes

**DOI:** 10.3390/cells9051172

**Published:** 2020-05-08

**Authors:** Stéphanie Maupetit-Mehouas, Chantal Vaury

**Affiliations:** GReD Institute, Université Clermont Auvergne, CNRS, Inserm, Faculté de Médecine, CRBC, 28 place Henri Dunant, TSA 50400, CEDEX 1, 63001 Clermont-Ferrand, France

**Keywords:** retroelements, transposable elements, piRNAs, DNA methylation, KRAB complex, silencing, germline, DRTS, Pilp

## Abstract

Transposable elements (TEs) are long-term residents of eukaryotic genomes that make up a large portion of these genomes. They can be considered as perfectly fine members of genomes replicating with resident genes and being transmitted vertically to the next generation. However, unlike regular genes, TEs have the ability to send new copies to new sites. As such, they have been considered as parasitic members ensuring their own replication. In another view, TEs may also be considered as symbiotic sequences providing shared benefits after mutualistic interactions with their host genome. In this review, we recall the relationship between TEs and their host genome and discuss why transient relaxation of TE silencing within specific developmental windows may be useful for both.

## 1. Introduction

Transposable elements (TEs) are genomic sequences having the ability to move from one location to another on the genome. They make up a large portion of eukaryotic genomes, as in Human where they account for almost 50% of the genome. The presence of TEs within genomes is dynamic. TEs tend to accumulate mutations and deletions leading the progressive extinction of the whole family in a species. Nonetheless, the reason TEs are widespread and persist in genomes is explained by the fact that at least some TEs remain active and have the capacity to regularly invade new species through horizontal transfers [1,2,3,4]. So, genomes display old, mostly inactive copies of TEs together with recent and mostly active copies of some families. TEs have long been considered as mutagenic agents that can generate insertional mutagenesis and chromosomal breaks upon mobilizations [5,6,7]. A long-lasting question has arisen so as to understand why old TE copies still persist in genomes if they are inactive. This is attributed to mutualistic interactions with their host resulting in shared benefit. On the one hand, TEs have been reported as evolutionary forces that shaped mammalian genomes. As a single and recent example, when exploring the evolutionary forces that shaped Muridae and Hominidae genomes, Thybert et al. showed that the ongoing expansion of the long interspersed nuclear element (LINE) retrotransposons appears to be associated with some gene cluster expansions in the different Muridae genomes [1]. Their data also suggest a model in which these repeats could have increased the susceptibility to rearrangements via nonallelic homologous recombination.

On the other hand, the host organism may take benefit of sudden remodeled regulatory programs due to TE integrations providing new enhancers, alternative promoters, or creating new exons that may add useful functions to the gene product [8,9,10,11]. Importantly, domestication of coding sequences of retrotransposons may create new genes. An iconic example is the independent capture of Env genes from Endogenous RetroViruses (ERV), called syncytins, useful for placentation [12]. During the last decade, studies able to estimate the global impact of TEs on gene regulatory networks have gained a lot with genome sequencing projects, the development of new bioinformatic tools, and whole-genome functional assays [13,14,15].

Beside the potential value of de novo integrations, the fact remains that TE amplification significantly contributes to genetic disease [16,17]. Conflict between TEs and their host genomes is then a critical factor limiting TE spreading. The genome has evolved sophisticated mechanisms to control TE mobilization. On their side, TEs have evolved a counter defense to escape from this host defense or limit its efficiency. This antagonistic host–TE coevolution is known as an arms race leading to a reciprocal adaptation [18,19,20].

In this review, we report the main mechanisms used by the host to restrain TE amplification and discuss developmental windows described in mice, drosophila, and plant reproductive tissues during which TE silencing is weakened allowing a transient TE transcription.

## 2. Genome Defense Plays a Crucial Role in Limiting TE Proliferation

Host genomes have developed mechanisms that reduce the cost of TE transposition, specifically in the germline where transposition has to occur to propagate within the population. These repressions occur both at the transcriptional (TGS) and post-transcriptional level (PTGS). This review is not intended to be a comprehensive review of these repressions, but rather a sampling of findings that have shed light on TE silencing and occasional desilencing in reproductive tissues. Such repressions employ DNA methylation, chromatin modifications including sequence specific transcriptional repression by Krüppel-associated box (KRAB) complexes, and small RNAs.

### 2.1. DNA Methylation

Cytosine DNA methylation is the main DNA methylation studied so far. It occurs mostly on the fifth carbon of cytosines (5-methylcytosine (5mC)) of symmetrical CpG dinucleotides. This DNA modification is widespread from bacteria to mammals but absent in some eukaryotes like *Drosophila melanogaster*, *Caenorhabditis elegans*, or fission yeast. During mammalian gametogenesis, DNA methyltransferases encoded by the *Dnmt3A* and *Dnmt3B* genes, and their catalytically inactive cofactor DNMT3L, mediate cytosine methylation of TEs. *Dnmt3L-/-* germ cells present a low level of methylation associated with a high TE expression [21]. Surprisingly, germ-cell-specific mutants of *Dnmt3B* have no effect on fertility, while *Dnmt3A* mutants are infertile with mild changes in TE methylation. DNMT3A and 3B are known as de novo DNMT, able to methylate hemi-methylated and unmethylated CpG. By contrast, the DNA methyl transferase called DNMT1 functions during DNA replication to copy the DNA methylation pattern from the parental DNA strand to the newly synthesized strand. Contrasting with *Dnmt3A-/-, 3B-/-*, a massive demethylation and derepression of the evolutionary young retrotransposons Intracisternal A particles (IAP) is observed in *Dnmt1*-null mutant Embryonic Stem Cells (ESCs) [22].

Another DNMT, DNMT3C, has been recently identified in mouse as a DNA methyltransferase specific to retrotransposons [23]. The *Dnmt3C* gene, which had been considered as a pseudogene, was revealed to originate from the duplication of *Dnmt3B* in the *Muroidea* lineage [22,24]. *Dnmt3C* expression is specific to male fetal germ cells and selectively methylates and represses the promoters of evolutionarily young transposable elements. How the Human genome lacks *Dnmt3C* copies with TEs is still an open question, but recent evolutionary analyses suggest that DNMT3A might be the enzyme carrying this function [25].

These DNMT studies converge on the notion that methylation targeting TEs limits TE transposition. In support of this, demethylating drugs activate TE transcription and unmethylated human L1 elements transpose at a higher rate in transfected cells than methylated L1 [23]. Moreover, cytosine DNA methylation has been proposed to limit the threats posed by genomic rearrangements due to recombination occurring between these dispersed homologous sequences. Methylation would restrain transposons from adopting a chromatin signature permissive for meiotic recombination [26]. Indeed, an increase of chromosomal rearrangements in human cancers is correlated with hypomethylation. Overall, Cytosine DNA methylation is reported as a key epigenetic modification required for gene regulation and is considered a host-control counteracting the threat of TEs [27]. As mammalian TE promoters are CpG rich, it has even been suggested that CG methylation has evolved for the specific purpose of defending the host genome against TE activity [27,28,29].

### 2.2. Sequence-Specific Transcriptional Repressors

Many studies have shown a clear correlation between Cytosine DNA methylation and the transcriptional inactive state of TEs. However, very little is known about how TEs are specifically targeted. Studies on the Krüppel-associated box (KRAB) domain containing zinc-finger proteins (KRAB-ZFPs) have brought some understanding.

KRAB-ZFPs are a rapidly evolving gene family, the root of which has been recently reported in a common ancestor of coelacanths and tetrapods [30]. KRAB-ZFPs are DNA binding factors containing an N-terminal KRAB domain followed by a variable array of C2H2-type zinc fingers [31,32]. Acting as transcriptional repressors, KRAB-ZFPs bind to DNA through their zinc finger domain. Via the KRAB domain, they recruit the corepressor KAP1 (also called TRIM28) which subsequently recruits epigenetic modifiers such as histone modifiers and DNA methyltransferases [33,34].

Characterization of KRAB-ZFP genomic targets has pointed out that many of them target and silence TEs. Most of our current knowledge comes from studies performed at very early stages of mammal development and in ESCs in which it was found that a large majority of KRAB-ZFPs associate with at least one subfamily of TEs. In mouse ESC, the Zinc Finger Protein ZFP809 targets TRIM28 to the primer binding site sequences (PBS) which are bound by specific tRNAs to prime MLVs and ERVs reverse transcription [34,35]. ZFP809 is then recognized as a stem-cell specific factor, targeting to silence a large subset of retroviruses and retrotransposons and participating in the intrinsic immune system of stem cells. The recruitment of KRAB-ZFPs may also occur to specific TE promoters or 3′ ends or be influenced by the age of the TE. In human embryonic stem cells (hES), KAP1 (TRIM28) represses a discrete subset of LINE1 (L1) elements corresponding to L1 having invaded the ancestral genome several millions of year ago [36]. If *KAP1* is knocked down in these cells, the expression of KAP1-bound L1 elements is induced, whereas the younger copies of these Human L1 are unaffected.

KRAB-ZFPs and TEs are thought to be locked in an evolutionary arms race, with new KRAB-ZFPs continuously emerging to cope with newly invading TEs [36]. This has been deduced from studies performed in a wide range of vertebrate species and reporting that the copy number of KRAB-ZFPs in these genomes correlates with the amount of LTR retroelements [37]. When investigating the evolutionary emergence of KRAB-ZFP genes in vertebrates and identifying their targets in the human genome, Imbeault and Trono found that many TEs whose activity has been lost for a long time since they invaded the genomes are still bound by KRAB-ZFP, suggesting that the arms race has not been the sole driver of selection and maintenance of KRAB-ZFP genes in mouse and human [30]. The data rather point out a domestication model in which some KRAB-ZFP co-opt TEs for the benefit of the host and may build a species-restricted layer of epigenetic regulation [28].

### 2.3. Transcriptional and Post-Transcriptional Silencing Mediated by Small RNAs

Small interfering RNAs accomplish silencing of genes targeted through RNA–RNA base pairing. In gonads, the major small RNA pathway involved in TE silencing is the PIWI-interacting RNA (piRNA) pathway. piRNAs are single strand RNAs of 23 to 32 nucleotides long that assemble in piRNA-induced silencing complexes (piRISCs) with PIWI proteins [38]. Cytoplasmic PIWI proteins are small RNA-guided nucleases that guide endonucleolytic cleavage of TE mRNAs. Nuclear PIWI proteins assemble silencing complexes on target genomic loci to mediate transcriptional silencing [39]. The piRNA-mediated silencing is active in the gonads of many species, including human, and has been discovered in *Drosophila* and mice in which the key steps of this mechanism have been elucidated. Regardless of which species, mutations affecting the pathway result in genome instabilities and sterility. In *Drosophila*, transcription of transposon rich loci called piRNA clusters, mostly located in pericentromeric regions, gives rise to long, single strand RNA precursors which are processed into piRNAs [40,41]. In the germline, piRNA clusters are mainly dual strand. Their transcription, including initiation and suppression of termination, requires the RDC complex made of Rhino (Rhi), Deadlock (Del), and Cutoff (Cuff) [42,43]. This complex recruits proteins required for transcription initiation within heterochromatin [44]. Rhino, Deadlock, and a recently identified gene CG13741/Bootlegger will then target the nuclear export factor 3 (Nxf3) to nascent piRNA precursors. After Cargo binding, Nxf3 achieves nuclear export of the unprocessed precursor transcripts. When in the cytoplasm, precursor transcripts accumulate in perinuclear nuage where piRNA processing occurs [45]. 

In the germline, piRNAs are amplified through a feed-forward RNA cleavage known as the ping-pong cycle [40]. This cleavage targets transcripts produced from both TEs and piRNA loci. It is achieved in the cytoplasm by the two PIWI proteins, AGO3 and Aub. TE transcripts being piRNA substrates of the ping-pong cycle, AGO3 and Aub are then actors of the post-transcriptional silencing of TEs. piRNAs amplified through the ping-pong cycle display specific signatures. When they are antisense to TE mRNAs, their 5′ end is rich in uridine (1U). When they are sense to TE mRNAs, they show an adenine bias at the 10th position (10A). The third PIWI protein identified in Drosophila, Piwi, is nuclear and accomplishes piRNA-mediated transcriptional silencing of TEs.

A similar piRNA amplification exists in mice. piRNAs act in complex with Argonaute proteins and silence TE expression by recognizing complementary RNAs. Disruption of the piRNA pathway in male mice leads to unsuppressed expression of certain TEs, which has been proposed to be at the origin of the sterility [46,47,48]. Most TE piRNAs originate from individual TE insertions and are amplified by the ping-pong cycle. Two groups of piRNAs can be distinguished according to the time of their expression during spermatogenesis (see below). In embryonic gonads, piRNAs resemble *Drosophila* piRNAs and silence TEs. Transposon silencing is then achieved thanks to MILI, a slicer-competent cytoplasmic Argonaute protein [47,48,49], and the nuclear MIWI2 proposed to recruit transcriptional silencing complexes over transcriptionally active TEs [50,51,52]. During the pachytene phase of meiotic prophase I, adult murine testes are highly enriched in piRNAs, but these piRNAs do not target TEs.

All these silencing pathways—DNA methylation, KRAB-ZFP epigenetic silencing, or small RNA targeting—act together to ensure TE silencing. Their multiple and complementary functions converge to repress all the families and each TE, be they old or recent genomic copies. They often mediate the deposition of similar chromatin modification, mostly histone tail modifications such as H3K9- and H4K20-trimethylation [53,54,55]. These mechanisms have been proposed to function in conjunction. Indeed, L1 elements are repressed even in the absence of both a functional piRNA pathway and DNA methylation in mitotic stages of spermatogenesis because of euchromatic repressive histone H3 dimethylated lysine 9 modifications cosuppressing L1 expression [56]. The network of silencing pathways may also work sequentially according to data reported by Castro-Diaz et al. Their data support a model in which new TEs would first be repressed by the DNA methylation-inducing small RNA-based mechanisms before KRAB-ZNP repressors are recruited [57].

## 3. Windows of Vulnerability to Transposition in Germ Cells

TEs have the possibility to occasionally evade the host controls. Indeed, several studies have reported that some TEs are highly expressed during short windows of germline development, a stage during which, paradoxically, TE control should be highly efficient to preserve genome integrity of the future organism and/or its progeny. These observations raise the possibility that replication cycles occur in these cells and lead to new genomic TE insertions. They also raise the possibility that TE transcripts produced at these specific stages play a cellular role during early development. Host genes and TEs might have a shared interest in such transient activations.

### 3.1. Resetting of Epigenetics Marks in Mammal Germ Cells

As proposed by Molaro and Malik [24], what we consider as germ cells are cells that are able to transmit information to the progeny such as those that become gametes but also those of the early zygote and the inner cell mass of the blastocyst. Most of our knowledge concerning germ cell formation in mammals comes from mouse models. After fecundation of the mature oocyte by a spermatozoid, the paternal and maternal pronuclei will then be submitted to numerous molecular events leading to the acquisition of the zygote epigenome and to the Zygote Genome Activation (ZGA). The embryo develops to the blastocyst stage at around 3.5 dpc (day post-coitum), and implants into the uterin mucosa. The embryo pursues its development and cell lineages differentiate to give rise to a newborn mouse at around 20 dpc.

**Figure 1:**
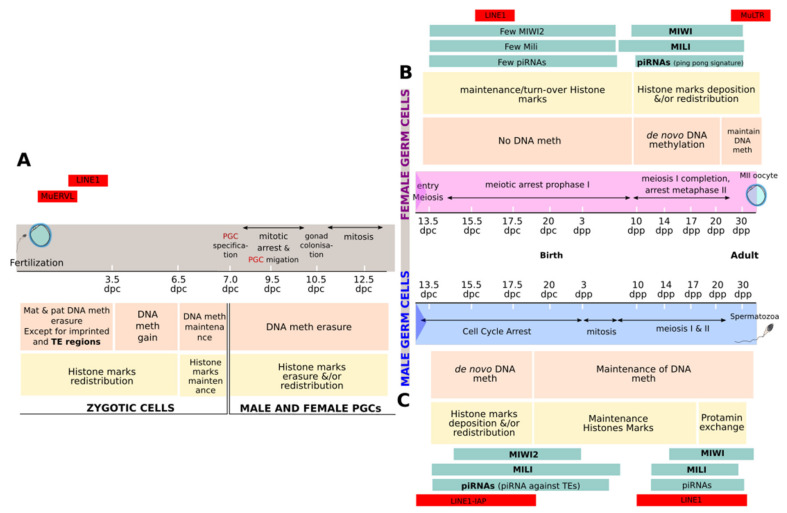
Schematic representation of transient transposable element (TE) relaxation during mouse germ cell development. From fertilization (**A**), maternal (mat) and paternal (pat) genomes undergo epigenetic reprogramming (DNA methylation (meth) resetting (orange) and chromatin remodeling (yellow)) that affects all genomic regions except imprinted regions and some TEs (red) until zygote implantation at 3.5 days post coïtum (dpc). After implantation, a new epigenetic landscape is established in the zygote. From 6.5 dpc, some cells of the zygote are specified as primordial germ cells (PGCs) and migrate through the genital ridge, the precursor of the gonad. A massive epigenetic reprogramming affects these PGCs until they differentiate into either the female (**B**, pink rectangle) or male germ line (**C**, blue rectangle). During germ cell differentiation, expression of numerous TEs (red rectangles) is observed and associated with the epigenetic reprogramming (DNA methylation in orange and histone modification in yellow rectangles). Actors of the piRNA pathway are differently expressed during these stages (green rectangle).

Formation of germ cells happens quickly after implantation of the blastocyst (3.5 dpc) in the uterine mucosa. Some cells emanate from the proximal epiblast and are specified into primordial germ cells (PGCs). They proliferate—then migrate and colonize the genital ridge—the future gonads of either male or female (Figure 1). From PGCs, spermatozoids and oocytes will differentiate with obvious differences.

In males, germ cells are blocked in mitosis at around 13.5 dpc until 3 dpp (day post-partum) where they resume their mitotic proliferation to give rise to a large pool of spermatogonia. At 8 dpp, male germ cells initiate meiosis and differentiate to ultimately give rise to spermatozoa [58].

In females, germ cells initiate meiosis at 13.5 dpc which is stopped at prophase I before birth. At 10 dpp, female germ cells pursue meiosis until a novel blockage at the metaphase II which gives rise to primary oocytes. The completion of meiosis of the mature oocyte will be achieved after fertilization by mature spermatozoids [58].

Two distinct global epigenetic reprogramming events take place within the germline that may affect the control of TEs. They occur after fertilization in all the zygotic cells during early embryo development at preimplantation stages, and in primordial germ cells of the developing embryo.

#### 3.1.1. Epigenetic Reprogramming during Early Embryo Development

The first epigenetic reprogramming event begins just after fertilization, when the methylated paternal pronucleus is decondensed through the exchange of the protamine by maternal histones. This is accompanied by a rapid paternal DNA methylation erasure, through a putative active process, which is still debated. At this stage, the maternal pronucleus maintains its DNA methylation levels, but a widespread redistribution of histones marks seems to be initiated [59,60]. From the 2-Cells-stages (2C-stages), maternal and paternal DNA undergo a passive demethylation process which erases all gametic DNA methylation until the blastocyst stage (3.5 dpc). Some genomic regions escape this loss of DNA methylation, including some TEs [61]. This specific DNA methylation maintenance and the hypothetic chromatin state may partly explain the absence of some TE desilencing during this developmental stage (Figure 1A).

Interestingly, specific de novo DNA methylation phases have been recently observed during this widespread DNA methylation erasure in human. Some genomic regions are specifically methylated including young and putatively active TEs (LINEs and SINEs). Actually, the molecular mechanism underlying this observation is not yet understood since this methylation is lost at later stages [62].

Nevertheless, this reprogramming step is a window of TE desilencing since almost 10% of the transcriptome from 2C-stage to blastocyst comes from TE transcription [63,64]. These TE reactivations are required for a proper embryonic development. This is the case of MuERV TE, giving one of the first transcripts expressed at a high level after fertilization (8 h), whose inhibition leads to an arrest of development at 2C-stages and alters ZGA [65].

Relaxation of TE silencing has also been reported in embryonic stem cells (ESCs). ESCs are pluripotent stem cells derived from the inner cell mass of blastocyst, a preimplantation stage embryo. Studies performed in ESCs in culture have nicely highlighted the potential impact of TEs on the developing embryo. In ESCs, the retrotransposon LINE1 which is the most abundant TE, and is still active in humans, is highly transcribed. Percharde et al. showed that these abundant LINE1 RNAs are nuclear in mouse ESCs where they recruit Nucleolin and KAP1/TRIM28 to repress Dux, the master activator of a transcriptional program specific to 2C-embryo [66]. In addition, LINE1 RNAs mediate binding of Nucleolin and KAP1 to rDNA and promote rRNA synthesis and ESC self-renewal. Accordingly, their depletion inhibits ESC self-renewal and induces the transition to a 2C-state. In embryos, it causes persistence of 2C-state and impairs ZGA. Thus, the specific transcription of LINE1 during this spatiotemporal window of mouse development is one of the actors orchestrating self-renewal of embryonic stem cells. Percharde et al. also stressed the fact that the role of LINE1 RNAs as chromatin-associated RNA avoids potential LINE1 retrotransposition and its associated detrimental effect. It has been proposed that the repeated and fast evolving nature of LINE1 might add robustness and adaptability to the regulation of early development. The same observation is made in cultured embryo whose RNA interference against its transcript leads to an arrest of early embryonic development [67,68].

#### 3.1.2. Epigenetic Reprogramming in Primordial Germ Cells of the Developing Embryo

Before sex specification, PGCs are subject to a global epigenetic remodeling characterized by a loss of CpG DNA methylation and remodeling of chromatin (Figure 1B,C). This epigenetic reprogramming leads to a complete “resetting” of the epigenetic memory arising from the parent and the establishment of sex-specific gametic identity. In mice, this reprogramming begins at the specification of the germ cells in male and female. As much as 70% of total CpG is methylated in “specified germ cells” of the epiblast at 6.5 dpc, compared to only 7% at 13.5 dpc [69,70]. This kinetics of demethylation is dependent of the specific characteristics of genomic regions, the demethylation process being slower for imprinted regions and retrotransposons [70,71]. These drastic losses of methylation are associated with a global remodeling of the chromatin characterized by a global increase/redistribution of some histone marks (H3K9me3 and H3K27me3) and a loss of others (H3K9me2). When germ cells are clearly differentiated into male and female, epigenetic reprogramming is then sex specific.

When female specificities are examined, it appears that female germ cells present a very low level of DNA methylation when blocked at prophase I of meiosis at 13.5 dpc. This low methylation is conserved until 10 dpp, where de novo DNA methylation will occur progressively with oocyte growth during folliculogenesis (Figure 1B). All this phenomenon is accompanied by a global histone mark modification. It is important to note that despite this important global loss of methylation during embryonic germ cell formation, no transcriptional burst is observed for all TEs except for a burst of LINE1 transcription at 16.5 dpc [70,72]. The same observation has been highlighted in fetal human germ cells where LINE1 and Alu clade transcripts have been found. These data suggest that, except for some of them, a control of numerous TEs is still effective during these spatiotemporal windows of epigenetic reprogramming that might be due to a chromatin-based repression [73].

In fetal female germ cells, inactivation of the piRNA pathway has no remarkable phenotype (females are fertile) but, according to current studies, some derepression of TEs is observed [74]. This suggests that the piRNA pathway has an impact on TE control during these developmental steps, uncoupled to sterility.

Later, in mature mouse oocyte, transcripts of LTR class III retrotransposons (such as the mouse transcript (MT) subfamily of MaLRs transposons (MT-LTR)) are extremely abundant [75]. It has been proposed that this transcription might be associated with specific functions of these cells at a later stage of germline development, such as oocyte attrition [49,50,76].

In male germ cells, de novo DNA methylation occurs quickly after 13.5 dpc to reach more than 40% of methylation at 16.5 dpc [70]. The level of methylation continues to rise during differentiation into mature sperm cells to reach around 90% [71]. Two waves of de novo methylation are observed during which some regions resist methylation. These regions mostly concern young TEs such as LINE1 and IAP elements. Histones are then replaced by protamines to induce a major chromatin compaction (Figure 1C). Transcriptomic studies have shown that numerous TE transcripts are upregulated during the formation of embryonic male germ cells. This expression decreases with the wave of DNA de novo methylation: 30% of all reads at 13.5 dpc, 20% at 16.5 dpc, and less than 15% in mature sperm cells. Some studies report that LINE1 ORF1 is expressed during fetal germ cells formation between 12.5 dpc and 16.5 dpc but not before. It is then downregulated after birth in spermatogonia [70,71]. These observations highlight the link existing between the epigenetic reprogramming occurring in male germline and relaxation of TEs.

The piRNA pathway plays an important function in the regulation of TE expression during embryonic male germ cell formation. The first expressed PIWI protein is MILI from 12.5 dpc to round spermatids, with some variation of its expression profile. Then, MIWI2 is expressed from 14.5 dpc to 3 dpp, a short window of male germ line development during which de novo DNA methylation occurs. MIWI2 is also involved in the establishment of a repressive chromatin state around these regions [77,78,79]. MILI and MIWI2 act both together and independently [80]. Since MILI or MIWI2 deficiency leads to a major TE derepression, it is proposed that demethylation during fetal germ cell formation leads to a relaxation of TEs, the transcripts of which will fuel the piRNA pathway using MILI and MIWI2 as main actors and increase the production of piRNAs [50,51,77,78]. The same mechanism seems to act in human fetal male germ cells, where elevated TE expression is detected and associated with a high expression of genes involved in the piRNA pathway [81].

After 3 dpp, MIWI2 ceases to be expressed and MILI is the only protein of the piRNA pathway still present. At preleptotene, its expression decreases to reach an undetectable level at pachytene (7 dpp to 12 dpp) [56]. Then, its expression increases again and remains stable until round spermatid. The third mouse PIWI protein, MIWI, is then expressed from pachytene stage to elongated spermatid (10 dpp to 30 dpp) [78]. A novel class of piRNAs, named pachytene piRNAs, is detected. The latter maps to unique sites in the genome with few exceptions for some repeated TEs suggesting that the main role of pachytene piRNAs is not to silence TEs [48,82].

Overall, studies performed in mammals point out that TE transcripts resulting from a loss of DNA methylation, specific epigenetic reprogramming, or weakness in the piRNA pathway during a short window of time may be used by the germline to i) increase piRNA production, which in turn re-establishes a tight silencing of TEs later on in the development; and ii) participate in gene regulation, orchestrating early development.

### 3.2. Weakness in the piRNA Pathway within the Dividing Cysts of Drosophila melanogaster Ovaries

In drosophila, primordial germ cells (PGCs) are the first cells that are cellularized in the syncytial embryo at its posterior pole. At the blastoderm stage (3.5 h post-fertilization), these cells stop mitosis in G2 of the cell cycle. Around embryonic stage 10, PGCs migrate, split into two groups, and coalesce with somatic gonadal precursor cells to form two gonads. In females, PGCs proliferate all along the larval stage to reach around 100 cells at the 3rd larval stage. In pupal ovaries, PGCs are in contact with somatic niche cells (called cap cells). In adult females, ovaries are formed by 15 to 16 structures called ovarioles that have two to three germline stem cells (GSCs) at their anterior pole which divide asymmetrically to produce a GSC and a cystoblast. The cystoblast will undergo four rounds of mitosis with incomplete cytokinesis to form a 16-cell germline cyst. This happens in the anterior part of the ovarioles, in a structure called germarium. GSCs divide continuously, pushing cysts posteriorly [83]. When the cysts of 16 interconnected cells move to the posterior of the germarium, one of them begins its differentiation into an oocyte. It initiates meiosis and arrests at prophase I until stage 13. The 16-cell cysts are then surrounded by somatic cells to give rise to the first egg chamber of the ovariole [84]. At stage 13, the oocyte progresses to metaphase I and arrests again at stage 14. Meiosis completion occurs during egg activation following fertilization.

**Figure 2:**
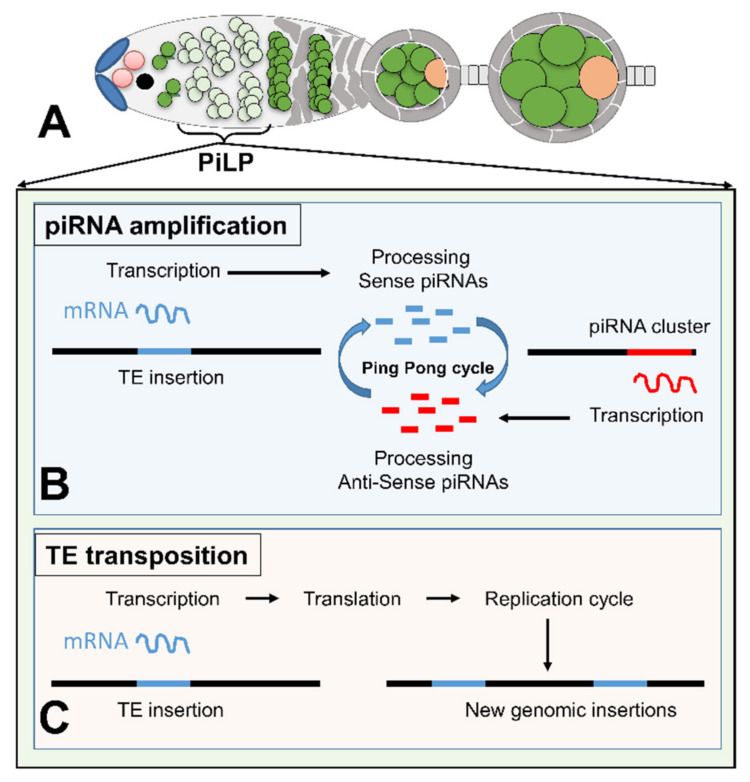
Model of the functional role of the “PiwiLess Pocket” (Pilp). (**A**) Schematic structure of a germarium and early egg chambers. Germline stem cells are in pink, the cystoblast in black, germ cells of the dividing cysts and nurse cells of the egg chambers in green with the Pilp indicated in light green, and the oocyte is in orange. The germinal cells are surrounded by somatic follicular cells (grey). (**B**) In the Pilp, TE transcripts resulting from a decrease of Piwi are processed into piRNAs that boost the ping-pong cycle through their complementarity to transcripts produced from piRNAs clusters (red). (**C**) In the Pilp, TE transcripts engage TEs in a new replication cycle leading to de novo genomic insertions.

Numerous studies performed on Drosophila TEs in the germline have reported that many of them have the capacity to be transcribed in this lineage, but their activity is blocked both at the transcriptional and post-transcriptional level by the piRNA pathway. Could this silencing be relaxed sometimes to allow some replication cycle to occur? Little is known about relaxation of TE silencing during embryonic, larval, and pupal development of Drosophila. Marie and Ronsseray reported that the P-element silencing may be occasionally relaxed due to an incomplete silencing established in the embryonic germ cells and stably maintained throughout development [85]. Besides such occasional and early TE derepression, it has been reported that a spatiotemporal window exists in the dividing cysts of adult ovaries during which TEs can escape from host silencing (Figure 2). A detailed analysis of the repression exerted on *Idefix*- and P-element-sensor transgenes along oogenesis indeed revealed that their repression is partially released within the germarium when the cystoblast undergoes mitotic divisions to form the interconnected 16-cell germline cyst [86]. Among the major factors required for the piRNA pathway, *aubergine (aub)* and *ago3* are both constantly expressed in the germline, including in the dividing cysts. Only *piwi* is poorly expressed in these germ cells, whereas its expression is clearly detected before and after mitotic cysts, in the GSC, in the cystoblast, and then from germarium 16-cell cysts to later stages. This spatiotemporal window during germline formation has been named the “PiwiLess Pocket” (Pilp). The specific lack of Piwi during this spatiotemporal window of drosophila germ cells is sufficient to allow TE transcription [87,88,89].

PIWI proteins have been reported to play a crucial role in germ cell specification and differentiation during early development. In Drosophila, removal of maternal PIWI affects germ cell specification by affecting the maintenance of pole plasm [87]. The existence of the Pilp is then puzzling and raises the question as to the consequences of such a piRNA pathway weakness on germline development. On both the host and TE point of view, dividing cysts are a very suitable stage for mobilizations (Figure 2). Their germline origin ensures that the new genomic insertions will be transmitted to the next generation. Whenever the mobilization is high and/or lethal for the future embryo, the mutational events will be narrowed to a cyst and the germinal stem cell will keep its potential to produce new nonmutated cysts [88]. Moreover, the production of TE transcripts may be useful to fuel the ping-pong cycle and promote piRNA amplification thanks to Aub and Ago3 that are correctly expressed in the Pilp [89]. Interestingly, this can be considered as a strategy of the host allowing TE transcription to better repress these mutagenic agents for the rest of the developing germline and future embryo (Figure 2).

### 3.3. Genes Responsible for Defense Against TEs Are Downregulated in the Vegetative Nucleus of Flowering Plants

Cell-specific bursts of TE transcriptional activity in a wildtype background of epigenetically silenced TEs are reported in Arabidopsis, maize, and rice pollen. It is named DRTS for Developmental Relaxation of TE Silencing. In Arabidopsis, the DRTS occurs in the vegetative nuclei (VN). Unlike animals, where the germline is established during early embryogenesis, plant sexual reproduction initiates with the formation of meiotic competent cells in adults. The pollen mother cell undergoes meiosis to give rise to four products of meiosis that each undergo two mitotic divisions. The first division is highly asymmetric and forms a binucleate pollen grain with a larger vegetative cell and a smaller germ cell (GC). The second division is performed by the small germ cell and leads to a pair of sperm cells (SCs). At the point of release from the anthers, mature pollen grains of *Arabidopsis thaliana* contain three germ cells of distinct cell type: two identical germ cells providing the paternal DNA inheritance to the zygote and the endosperm. The vegetative nucleus does not contribute to the zygotic DNA, but it controls the delivery of sperm. Its DNA is in a decondensed state compared to the compact sperm nuclei [90].

Although an epigenetic silencing represses TEs throughout most plant development, a transient reactivation of TEs has been found in mature pollen of flowering plants. In *Arabidopsis thaliana,* both expression and transposition only occur in the VN and is correlated with a lack of chromatin remodeling ATPase Decrease in DNA Methylation 1 (DDM1), one of the main actors involved in TE silencing throughout plant development and found accumulated in the SCs [91]. It was further found that euchromatic TEs in the pollen VN undergo DEMETER-dependent DNA demethylation [92].

Differing from animal piRNAs, two types of plant gametophytic small RNAs assure repression of TEs and transgenerational inheritance of heterochromatin identity. Together with the RNA-directed DNA methylation (RdDM) pathway, 24-nt long RNAs assure TE transcriptional silencing (TGS). If reactivated, TEs can produce 21/22-nt long RNAs that assure a post-transcriptional silencing (PTGS) or, if loaded onto AGO6, also direct TGS [93]. A careful analysis of the mature sperm indicated that 24-nt small RNAs are lacking whereas 21-nt RNAs dramatically increase for several TEs.

**Figure 3:**
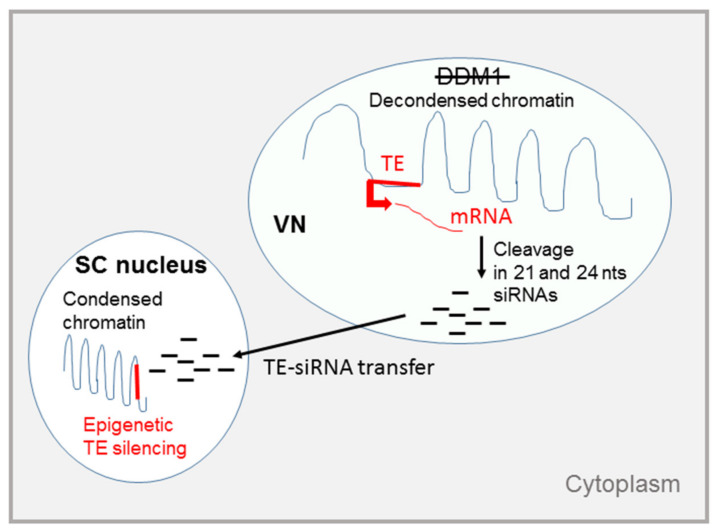
The potential role of siRNAs in the Arabidopsis male gametophyte. In the VN, the chromatin is in a decondensed state due to a lack of the chromatin remodeling protein DDM1. The transcription of TEs is allowed and gives rise to mRNAs that are cleaved in 21- and 24-nt siRNAs. These siRNAs may get out of the VN and enter the neighboring sperm cell where they direct DNA methylation of complementary TEs or genes. DNA is in blue, TEs and their mRNAs are in red. Cell-to-cell movement is indicated by an arrow.

Substantial knowledge gaps remain to be filled to fully appreciate the functional role of these small RNAs during the epigenetic reprogramming that has been observed in Arabidopsis meiocytes, endosperm, and nurse cells of gametophytes. Despite its potential danger, this cell-type specific epigenetic reprogramming could serve as a mechanism revealing TE presence to the germline, ensuring their control after fertilization. The 21-nt small RNAs accumulate in the VN and can migrate to the associated SC to reinforce silencing (Figure 3) [94]. Supporting that the DRTS function in companion cells is to reinforce TE silencing in gametes, depletion of the demethylase DEMETER in the VN not only leads to an increase of DNA methylation in the VN, but also to a reduced methylation of TEs in the sperm cells. Overall, these data indicate that the very strict reactivation of TEs in the VN contributes to transgenerational TE silencing [92].

In addition to this, the potential role of DRTS could be to help TEs to evade long-term heterochromatic silencing. Releasing their silencing offers TEs the opportunity to start the replication cycle. Finally, although specific to TE-silencing and heterochromatin maintenance, a role of DRTS in regulating specific genes important in plant development has also been envisioned. In support of this, the endosperm-imprinted genes required for seed development are sensitive to TE activity [95].

## 4. Conclusions

A tight silencing of TEs is exerted through multiple and complementary mechanisms involving DNA methylation, transcriptional repressors, or small RNA production. However, TE-epigenetic transcriptional derepression occurs at specific stages during the normal development of wildtype organisms. In a selfish point of view, TEs could take this opportunity of chromatin reorganization required for cellular functions to transpose. However, this relaxation has been reported under strict developmental control in the germline of wildtype animals and plants, which rather suggests a programmed TE reactivation playing a functional role. The mechanisms allowing developmental precision of TE desilencing are just being elucidated. It is hard to estimate yet the significance and importance of this reactivation. However, host genes and TEs could share a mutual interest of such releases of control. Several examples are reported in this review, but one can anticipate that the list is far to be exhaustive. These TE reactivations during germline development add a new level of complexity in the relationship between TEs and their host genome and its potential trans-generational impact.
